# Mitochondrial-Targeted Antioxidant MitoQ-Mediated Autophagy: A Novel Strategy for Precise Radiation Protection

**DOI:** 10.3390/antiox12020453

**Published:** 2023-02-10

**Authors:** Xingting Bao, Xiongxiong Liu, Qingfeng Wu, Fei Ye, Zheng Shi, Dan Xu, Jinhua Zhang, Zhihui Dou, Guomin Huang, Hong Zhang, Chao Sun

**Affiliations:** 1 Department of Medical Physics, Institute of Modern Physics, Chinese Academy of Sciences, Lanzhou 730000, China; 2 Advanced Energy Science and Technology Guangdong Laboratory, Huizhou 516000, China; 3 Key Laboratory of Heavy Ion Radiation Medicine of Gansu Province, Lanzhou 730000, China; 4 Key Laboratory of Heavy Ion Radiation Biology and Medicine of Chinese Academy of Sciences, Lanzhou 730000, China; 5 College of Life Sciences, University of Chinese Academy of Sciences, Beijing 101408, China

**Keywords:** MitoQ, PMMP, autophagy, radioprotection, energy phenotype

## Abstract

Radiotherapy (RT) is one of the most effective cancer treatments. However, successful radiation protection for normal tissue is a clinical challenge. Our previous study observed that MitoQ, a mitochondria-targeted antioxidant, was adsorbed to the inner mitochondrial membrane and remained the cationic moiety in the intermembrane space. The positive charges in MitoQ restrained the activity of respiratory chain complexes and decreased proton production. Therefore, a pseudo-mitochondrial membrane potential (PMMP) was developed via maintenance of exogenous positive charges. This study identified that PMMP constructed by MitoQ could effectively inhibit mitochondrial respiration within normal cells, disrupt energy metabolism, and activate adenosine 5′-monophosphate (AMP)-activated protein kinase (AMPK) signaling to induce autophagy. As such, it could not lead to starvation-induced autophagy among tumor cells due to the different energy phenotypes between normal and tumor cells (normal cells depend on mitochondrial respiration for energy supply, while tumor cells rely on aerobic glycolysis). Therefore, we successfully protected the normal cells from radiation-induced damage without affecting the tumor-killing efficacy of radiation by utilizing selective autophagy. MitoQ-constructed PMMP provides a new therapeutic strategy for specific radiation protection.

## 1. Introduction

RT is one of the current first-line treatment options for cancers, with over 50% of all cancer patients being treated with RT [1,2]. However, RT can induce short-term and long-term toxicity. For instance, the exposure of brain tissue to therapeutic radiation is related to various adverse outcomes, including long-term neurocognitive sequelae, cognitive impairment, and endocrine dysfunction [3,4]. Thus, eliminating tumors while selectively protecting normal tissues is challenging for tumor radiotherapy. However, no radio-protective agents are clinically available for brain tumors, except for memantine hydrochloride, which is in clinical recruiting status [5]. Additionally, the only US Food and Drug Administration (FDA)-approved drug, amifostine, provides radioprotection in head and neck cancer and is unsuitable due to side effects and incomplete protection [6]. Many antioxidants ameliorate or prevent the side effects of RT, but some even potentially promote cancer development and metastasis in mice models [7,8,9,10]. Thus, unmet medical demand for safer and more effective radio-protective agents persists.

MitoQ, a mitochondria-targeted antioxidant, is developed to penetrate the blood–brain barrier (BBB) and neuronal membranes and has a good neuroprotective effect [11,12]. In our previous study, MitoQ induced autophagy by affecting mitochondrial respiration by constructing PMMP [13]. Autophagy is an intracellular “self-digestion” physiological process that relies on lysosomes to degrade cytoplasmic unnecessary or dysfunctional components [14]. Under normal physiological states, the binding of adenosine triphosphate (ATP) to AMPK suppresses the kinase activity. When energy starvation appears in cells, the ATP levels decrease, and the AMP levels increase. An important “energy sensor”—AMPK in eukaryotic cells is phosphorylated. The activated AMPK causes defective mammalian target of rapamycin (mTOR) signaling by inhibiting the phosphorylation of mTOR, boosting the formation of autophagosomes and autophagy flux [15,16,17]. Nature Chemical Biology reported that inducing autophagy within ischemic brain cells by small molecule compounds can reduce the expression of apoptotic factors, clear damaged organelles in time, and degrade and reuse accumulated long-lived proteins to supply energy for ischemic brain cells quickly, which is conducive to maintaining cell homeostasis and protecting cells [18]. The α1-antitrypsin mutation can lead to hepatic endoplasmic reticulum stress, severe inflammatory response, and liver damage and carcinogenesis. It is reported that autophagy effectively removes the mutated α1-antitrypsin in liver cells, relieves the stress state of the liver, and has a potent liver protection effect [19]. Therefore, autophagy ensures cell integrity, maintains effective cell function, helps cells survive the crisis, and promotes cell survival in extreme cases [20]. Thus, if autophagy can be selectively induced among radiated normal cells, normal tissue can be effectively protected during RT.

Eukaryotic cells primarily rely on oxidative phosphorylation (OXPHOS) of the mitochondrial respiratory chain for energy supply [21]. The electrons carried by nicotinamide adenine dinucleotide (NADH) and flavin adenine dinucleotide (FADH_2_) are utilized as fuel and offered for the molecular oxygen by the mitochondrial inner membrane respiratory chain. This generates a proton gradient with proton pumping into the mitochondrial intermembrane space through the respiratory chain complexes I, III, and IV. The dissipation of the established proton gradient using complex V generates ATP [22]. Thus, the energy supply in normal cells originates from mitochondria, and the proton gradient inside the mitochondrial intermembrane space is the driving force maintaining the energy synthesis of cells. However, in the 1950s, Otto Warburg discovered a characteristic of energy metabolism distinguishing tumor cells from normal cells through high levels of glycolysis. Warburg believed that tumorigenesis led to mitochondrial respiratory dysfunction in tumor cells. Tumor cells reprogram their energy metabolism by initiating mitochondria-independent energy supply pathway: aerobic glycolysis called the “Warburg effect” [23,24,25] to maintain normal intracellular ATP and NADH levels to ensure the need for malignant proliferation. This fact has been applied in positron emission tomography (PET) depending on 18F-fluorodeoxyglucose (FDG). The degree of glucose uptake of a malignancy as imaged by FDG-PET is associated with histologic measures of tumor differentiation [24]. In summary, significant differences in energy metabolism exist between normal and tumor cells. Therefore, if the mitochondria-dependent energy supply pathway is targeted, leading to normal cell starvation, autophagy can be selectively induced within normal cells. However, it is ineffective among tumor cells independent of mitochondria and relies primarily on glycolysis for energy supply. Here, we investigated the protective effect of MitoQ-constructed PMMP on normal cells during irradiation by selectively inducing autophagy in normal cells. In contrast, tumor cells are not protected due to the absence of autophagy.

## 2. Methods and Materials

### 2.1. Cell Culture

Human Astrocytes (HA) were purchased from ScienCell^TM^ and cultured in Astrocyte Medium (ScienCell^TM^, Cat No.1801, San Diego, CA, USA) with 2% fetal bovine serum (ScienCell^TM^, 0010), 1% penicillin/streptomycin (ScienCell^TM^, 0503), and 1% astrocyte growth supplement (ScienCell^TM^, 1852), in 5% CO_2_ and at 37 °C. The culture vessels were coated with poly-L-lysine stock solution (ScienCell^TM^, 0413) at 2 μg/cm^2^. HA was utilized within 10 generations, authenticated using short tandem repeat (STR, ScienCell^TM^), and routinely tested for *Mycoplasma*. Human glioblastoma cell line A172 cells were obtained from BeNa Culture Collection (BNCC) and cultured in RPMI 1640 medium (Meilunbio, MA0215-2, Liaoning, China) using 10% fetal bovine serum (TransGen Biotechnology, FS101-02, Beijing, China), in 5% CO_2_ and at 37 °C. A172 cells were used within 15 passages from frozen stocks, authenticated using STR (BNCC), and were routinely evaluated for *Mycoplasma*.

### 2.2. Animals

Six-week-old male BALB/c nude mice were utilized for high performance liquid chromatography–mass spectrometry (HPLC-MS) and matrix-assisted laser desorption/ionization time-of-flight mass spectrometry (MALDI-TOF-MS) imaging. The mice weighed approximately 20 g and were purchased from Beijing Vital River Laboratory Animal Technology Co., Ltd. (Beijing China). In addition, six-week-old male BALB/c nude mice bearing luciferase-positive U87MG orthotopic brain tumors, weighing approximately 20 g, were procured from Wuhan Servicebio Biotechnology Co., Ltd. (Wuhan China). All the mice were housed under specific pathogen-free (SPF) conditions in facilities having freely available food and water. All animal procedures strictly followed the animal experimentation regulations in China and were approved by the Animal Ethics Committee of the Institute of Modern Physics of the Chinese Academy of Science (No. 2022(06)).

### 2.3. Polymerase Chain Reaction (PCR) Array Expression Profiling

The Human Signal Transduction Pathway Finder™ RT^2^ Profiler™ PCR Array (Qiagen) determined the expression levels of 50 genes associated with glycolysis and the tricarboxylic acid (TCA) cycle within HA and A172 cells. Microarray data were normalized against the house-keeping genes by evaluating the ΔCt for each gene representative of glycolysis and TCA in the plate. Data were analyzed using the RT^2^ PCR array data analysis web portal version 3.5.

### 2.4. Agilent Seahorse XF Technology

The Agilent Seahorse XF technology was used to analyze the cell energy phenotype of HA and A172 cells using the Agilent Seahorse XFp Cell Energy Phenotype Test Kit (Agilent, 103275-100, Santa Clara, CA, USA) based on the instructions. HA and A172 cells were grown in the Agilent Seahorse XFp Cell Culture Miniplate and cultured overnight. The Agilent Seahorse XFp Sensor Cartridge was hydrated using Agilent Seahorse XF Calibrant at 37 °C in a non-CO_2_ incubator overnight. The Agilent Seahorse Sensor Cartridge, Calibrant, and Miniplate were obtained from the Agilent Seahorse XFp FluxPak (Agilent, 103022-100). The assay medium was prepared by supplementing Agilent Seahorse XF Base Medium (Agilent, 102353-100) with 1 mM pyruvate (Sigma, S8636, St. Louis, MO, USA), 2 mM glutamine (Sigma, G8540), and 10 mM glucose (Sigma, G8769). The cell culture medium of HA and A172 were replaced with the assay medium and cultured in a non-CO_2_ incubator for 1 h. Oligomycin and cyanide p-trifluoromethoxylphenyl-hydrazone (FCCP) from the Agilent Seahorse XFp Cell Energy Phenotype Test Kit were combined to develop a stressor mix loaded into every port A of the hydrated sensor cartridge. The Agilent Seahorse XF Cell Energy Phenotype test was run using the Agilent Seahorse XFp Analyzer (Agilent,102745-100). The data were analyzed with the Agilent Seahorse XF Cell Energy Phenotype Test Report Generator.

### 2.5. HPLC-MS Analysis

The enrichment of MitoQ (Vosun Chemical, 444890-41-9) in mitochondria in vitro and the concentrations in brain and blood in vivo were determined using an EVOQ Qube LC-TQ system (Bruker, Germany) as in our previous study [13]. For measuring the MitoQ enrichment inside mitochondria in vitro, the cells were harvested and divided into two equal parts after treatment with MitoQ for 2 h, one for the homogenate of the whole cell and the other for the homogenate of the mitochondria. A mitochondrial extraction kit (Beijing Solarbio, SM0020, Beijing, China) was used to isolate the mitochondria. All the homogenates were extracted twice utilizing the mixture of methylene chloride and methanol at a 2:1 volume ratio, including the 2 mM butylated hydroxytoluene (Sigma-Aldrich, W218405). After drying, the residue was dissolved in cold methanol with 2 mM butylated hydroxytoluene.

For measuring the MitoQ in brain and blood in vivo, six-week-old male BALB/c nude mice were randomly divided into three groups (*n* = 5): control, intraperitoneal injection (i.p.), and intragastric administration (i.g.) groups. The i.p. and i.g. groups were administered intraperitoneally and intragastrically with MitoQ (5 mg/kg) for three days, respectively. Mice in the control group were administered intraperitoneally with saline for three days. Mice were sacrificed after treatment with MitoQ for 1 h on the last day, and blood and brain samples were collected to determine MitoQ. All the blood and brain samples were homogenized and processed as described above. Moreover, the gradient elution time was 6 min per sample, and MitoQ depicted a retention time close to 3 min.

### 2.6. Determination of Mitochondrial Membrane Potential (MMP) 

Changes in the MMP were identified using JC-1 Mitochondrial Membrane Potential Assay Kit (Beyotime, C2006). The treated cells were incubated with a JC-1 working solution for 20 min at a 37 °C, 5% CO_2_ incubator. Then, the cells were washed twice with 1×JC-1 staining buffer and analyzed by a CytoFLEX flow cytometer (Beckman, South Kraemer Boulevard Brea, CA, USA). 

### 2.7. Detection of Respiratory Chain Complex Activities

HA and A172 cells were treated using MitoQ for 2 h and collected for isolating the mitochondria through a Mitochondrial extraction kit. After estimating the protein concentration of isolated mitochondrial samples, the respiratory chain complex activities were detected using the Mitochondrial Complex I Activity Colorimetric Assay Kit (Abcam, ab287847, Cambridge, UK) and the Mitochondrial Complex III Activity Assay Kit (Abcam, ab287844) based on the instructions provided with the reagent kits.

### 2.8. Measurement of H^+^/2e

Mitochondria were extracted from HA and A172 cells through the Mitochondrial extraction kit. Isolated mitochondria were suspended within a solution with 0.25 M sucrose (Sigma, S9378), 1 mM ethylenebis (oxyethylenenitrilo) tetraacetic acid (EGTA), and 5 mM Tris, pH 7.4 at 0 °C. The proton pump rate (PPR) underwent the K_3_Fe(CN)_6_ pulse method as in our previous study [13]. Electron transport rates and proton ejection were determined using 557 double-beam spectrophotometers (PerkinElmer, Waltham, MA, USA) and PHM84 fast-responding pH electrode system (Radiometer Medical, Copenhagen, Denmark).

### 2.9. Untargeted Metabolomics Based on Ultra-High Performance Liquid Chromatography-Tandem Time-of-Flight Mass Spectrometry (UHPLC-Q-TOF MS)

The cells were treated using MitoQ, and after collection, the cells were washed with pre-cooled phosphate buffered saline (PBS) three times and removed from the supernatant. Pre-cooled methanol: acetonitrile: water (2:2:1, *v/v*) solution was added to each sample to precipitate protein. The samples were pulverized by the ultrasonic wave at 0 °C for 30 min, incubated at −20 °C for 10 min, and then centrifuged at 4 °C, 14,000× *g*, for 20 min. The supernatants were transferred into new 1.5 mL tubes and dried inside a centrifugal vacuum evaporator, then reconstituted using a 100 μL acetonitrile: water (1:1, *v/v*) mixture for further analysis. The metabolites in the samples were detected with Shanghai Applied Protein Technology Co., Ltd. (Shanghai, China) using UHPLC-Q-TOF MS. which is a 1290 Infinity Ultra-high Performance Liquid Chromatography system (Agilent, Santa Clara, CA, USA) coupled with AB Triple TOF 6600 Mass Spectrometer (AB SCIEX, Radio Road Redwood City, CA, USA). 

### 2.10. Western Blot Analysis

The cells were lysed in RIPA buffer (Solarbio, R0010). The protein concentration was determined using the BCA Protein Assay Kit (Meilunbio, MA0082). Equivalent amounts of protein were separated through SDS-polyacrylamide gels and electroblotted onto PVDF membranes (Bio-Rad, 1620177). After blocking using a 5% BSA Blocking Buffer (Solarbio, SW3015), the membranes were incubated with the primary antibody at 4 °C overnight. The antibodies used were AMPKα Antibody (Cell Signaling Technology, 2532, Danvers, MA, USA), Phospho-AMPKα Rabbit mAb (Cell Signaling Technology, 2535), mTOR Antibody (Cell Signaling Technology, 2972), Phospho-mTOR Antibody (Cell Signaling Technology, 2971), LC3B (E5Q2K) Mouse mAb (Cell Signaling Technology, 83506), SQSTM1/p62 Antibody (Cell Signaling Technology, 5114), and β-Actin Antibody (Cell Signaling Technology, 4967). Finally, the relevant protein was visualized through staining with an appropriate HRP-conjugated secondary antibody for 1 h and then enhanced with chemiluminescence. The expression of the protein was quantified using ImageJ software.

### 2.11. Transmission Electron Microscopy (TEM)

The ultrastructural analysis of autophagy was observed using TEM. The samples were fixed using a 3% glutaraldehyde solution (Sigma-Aldrich, G5882) for 24 h at 4 °C and post-fixed in 1% osmium tetroxide (Sigma-Aldrich, 75632) at 4 °C for 1 h. The cells were washed twice, dehydrated in successive ethanol baths, treated with two baths of propylene oxide, and progressively impregnated and embedded within Epon-Araldite resin (Electron Microscopy Sciences, 14900, Hatfield, PA, USA). Ultrathin sections of 50 nm thickness were acquired through a Leica Ultracut (Leica Microsystems, Wetzlar, Germany; EM UC6) and stained using uranyl acetate and lead citrate. The autophagosomes were observed through JEM-1220 TEM (JEOL, Tokyo, Japan).

### 2.12. Observation of Autophagosome and Autophagy Flux

The high content imaging system was used to visualize autophagic vacuoles within live cells. HA and A172 cells were grown in ViewPlate-96 Black, clear bottom, TC-treated (PerkinElmer, 6005182), and cultured overnight. The treated cells were stained using Cell Meter™ Autophagy Assay Kit (AAT Bioquest, 23002) and Hoechst 33,342 (Meilunbio, MA0126) based on the manufacturer’s instructions. The images were acquired and analyzed through the Operetta CLSTM High content imaging system (PerkinElmer, Waltham, MA, USA). 

Confocal microscopy was used to visualize and monitor autophagy flux in live cells. HA and A172 cells were seeded into a 35 mm confocal dish (NEST, 801001) and cultured overnight. Then, the cells were treated with MitoQ for 2 h. After that, cells were treated using the nuclear dye Hoechst 33,342, Autophagy Assay Kit (green), and LysoBrite Red (AAT Bioquest, 22645). The confocal images were acquired through Laser confocal microscopy (LSM700, Zeiss, Germany).

### 2.13. In Vitro Cell Proliferation Analysis

The Cell Counting Kit-8 (CCK8, Meilunbio, Dalian, China, MA0218) and 5-ethynyl-2′-deoxyuridine (EdU, Meilunbio, MA0425) labeling assay were used to measure cell proliferation based on the manufacturer’s protocols. In brief, for the CCK8 assay, HA and A172 cells were seeded inside the 96-well microplates and cultured overnight. After incubation with MitoQ for 2 h, the cells were radiated using X-rays at doses of 4 Gy or carbon ion at 2 Gy. Then, 10 μL of CCK-8 reagent was added to each well after 24 h and incubated for 2 h. The optical density (OD) was determined at 450 nm through a multi-plate reader (Tecan Infinite M200, Männedorf, Switzerland). 

For the EdU labeling assay, HA and A172 cells were seeded into a 35 mm confocal dish and cultured overnight. After treatment using MitoQ for 2 h, the cells were radiated with 4 Gy X-rays or 2 Gy carbon ion. Based on the manufacturer’s instructions, the EdU kit was utilized for assessing cell proliferative ability after irradiating for 24 h. Hoechst 33,342 (nuclear staining) was utilized to counterstain cells. The images were captured with Laser confocal microscopy.

### 2.14. MALDI-TOF-MS Imaging Mass Spectrometry Analysis

MALDI-TOF-MS imaging is used for label-free bioanalysis of the spatial distribution of pharmaceuticals, biomolecules, and other molecules from a tissue section [26]. The autoflex speed™ MALDI-TOF-MS imaging (Bruker, Bremen, Germany) was utilized to reveal the quantitative distribution of MitoQ across a tissue section (8 μm) having high spatial resolution from BALB/c nude mice injected with 5 mg/kg/day MitoQ intraperitoneally for three consecutive days. 2,5-Dihydroxybenzoic acid (DHB, Bruker, 8201346, 30 g/L) with 1% trifluoroacetic acid was utilized as the matrix for MALDI-TOF-MS imaging analysis.

### 2.15. In Vivo Imaging of Orthotopic Glioma Using Bioluminescence Imaging (BLI) and Magnetic Resonance Imaging (MRI)

The tumor luciferase expression determines the tumor size through an in vivo imaging system (PerkinElmer IVIS Lumina LT Series III, Waltham, MA, USA). The mice were injected intraperitoneally using 150 mg/kg of D-luciferin (PerkinElmer, Waltham, MA, USA) and anesthetized with vaporized isoflurane. The mice were stratified into sham, X-ray, and X-ray + MitoQ groups depending on the tumor luciferase expression (*n* = 5). The sham and X-ray groups received an i.p. injection of 200 μL saline for four consecutive days. The X-ray + MitoQ group was intraperitoneally injected using MitoQ (10 mg/kg/day) for four days. The X-ray and X-ray + MitoQ group received 16 Gy X-ray whole brain radiation on the third day 2 h after administration.

For MR imaging, T2-TSEI and T2-FLAIR MR images were acquired 24 h and seven days post-X-ray radiation, respectively, through a 1.0-tesla small animal magnetic resonance scanner (XGY OPER 1.0). The coronal images were obtained with the following parameters: FOV (field of view) = 4.0 × 4.0 cm, TR/TE = 4000/91 ms for T2-TSEI images, and TR/TE = 7000/104 ms for T2-FLAIR images, Matrix = 160 × 154, and Slice Thickness = 1.3 mm. The sagittal and horizontal images were obtained with the following parameters: FOV = 5.0 × 5.0 cm, TR/TE = 5000/104 ms for T2-TSEI images, and TR/TE = 7500/101 ms for T2-FLAIR images, Matrix = 192 × 182 for T2-TSEI images and Matrix = 192 × 198 for T2-FLAIR, with Slice Thickness = 1.2 mm.

### 2.16. Histomorphological and Terminal dUTP Nicked End LABELING (TUNEL) Assessments of Brain

We used hematoxylin and eosin (H&E) staining to analyze damages caused by X-rays to normal brain tissue to determine the protective effects of MitoQ on normal brain tissue during X-ray radiotherapy. All the mice were sacrificed by cervical dislocation, and then the brain tissues were embedded inside Tissue-Tek optimal cutting temperature (OCT) Compound (SAKURA, 4583) and sectioned (12 μm). The 12 μm cryosections were stained using H&E and examined to observe histomorphological features through the Pannoramic 250 Flash digital microscopes (P250 Flash digital microscopes; 3DHISTECH, Budapest, Hungary). TUNEL assay was utilized to detect cell death-associated DNA fragmentation (3′-OH DNA termini) on the tissue sections [27]. The tissue sections were stained based on the instructions provided by the Fluorescein (FITC) Tunel Cell Apoptosis Detection Kit (Servicebio, G1501). Then, the slides were incubated with DAPI (Invitrogen) for 10 min and observed under P250 Flash digital microscopes.

### 2.17. Statistical Analysis

Statistical analysis was conducted using GraphPad Prism. The statistical analyses performed for different data are demonstrated in each figure legend, and the data were considered statistically significant if *p* < 0.05.

## 3. Results

### 3.1. Cell Energy Phenotype of HA and A172 Cells

**Figure 1:**
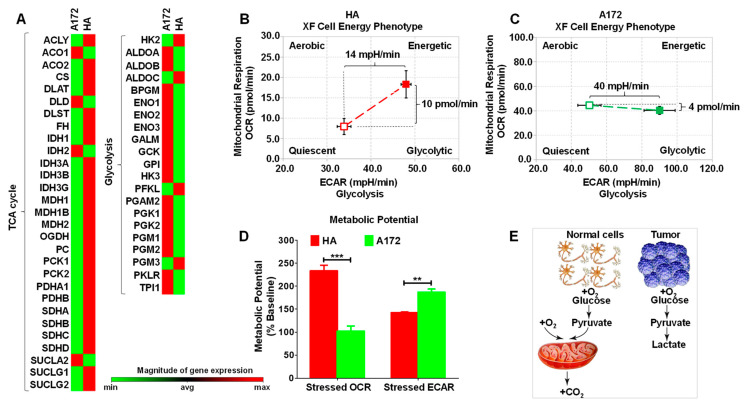
Cell energy phenotype of HA and A172 cells. (**A**) Human Signal Transduction Pathway Finder PCR Array was used to evaluate the expression of 50 essential genes related to glycolysis and the TCA cycle. Red indicates high expression levels, whereas green indicates low expression levels (*n* = 3). The Agilent Seahorse XFp Cell Energy Phenotype Test Kit was used to assess that normal astrocyte HA tends to supply energy through mitochondrial respiration (**B**). In contrast, glioma cell A172 tends to provide energy through glycolysis (**C**). (**D**) The metabolic Potential of HA and A172 cells is the ability of the cell to meet energy demand through respiration or glycolysis. (**E**) Schematic diagram of the different cellular energy phenotypes of normal and tumor cells. All the data were presented as mean ± SEM. Error bars represent SEM, and statistical significance between groups was analyzed using an unpaired *t*-test. ** *p* < 0.01; *** *p* < 0.001.

In normoxia, fully differentiated tissues utilize OXPHOS. However, the most common metabolic hallmark of malignant tumors, i.e., the “Warburg effect,” maintains a malignant tumor phenotype (Figure 1E) [28]. A heat map revealed that most glycolysis genes were up-regulated in A172 cells compared to HA cells, and genes associated with the TCA cycle were down-regulated (Figure 1A). Agilent Seahorse XF technology was further used to evaluate the cell energy phenotype of HA and A172 cells [29]. Based on the instructions, metabolic potential represented the percentage increase in stressed oxygen consumption rate (OCR) relative to the baseline OCR and stressed extracellular acidification rate (ECAR) relative to the baseline ECAR. It measures the ability of the cells to meet energy demand through mitochondrial respiration or aerobic glycolysis. Under the stress conditions induced by oligomycin and FCCP, ECAR increased by 40 mpH/min and OCR decreased by 4 pmol/min in A172 cells, while ECAR increased by 14 mpH/min and OCR did by 10 pmol/min in HA cells (Figure 1B,C). Compared to HA, the stressed ECAR/baseline ECAR ratio was significantly increased and the stressed OCR/baseline OCR ratio was significantly decreased in A172 cells (Figure 1D). The results depicted that HA cells meet energy demand primarily by mitochondrial respiration, while A172 cells meet energy needs primarily through aerobic glycolysis. Our results are consistent with the previous studies [28,30].

### 3.2. MitoQ Was Selectively Enriched in Mitochondria in HA Cells Higher than That in A172 Cells

**Figure 2:**
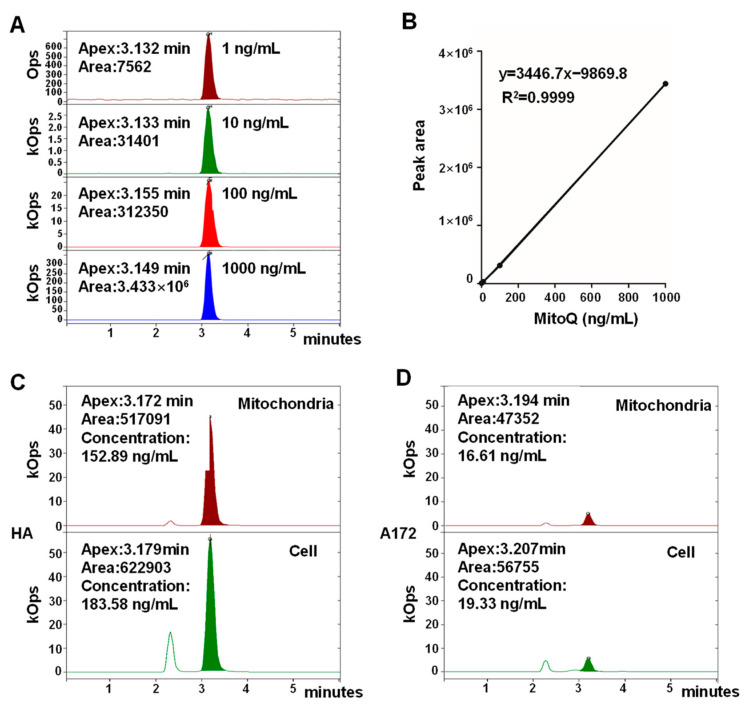
Quantitative analysis of MitoQ enrichment in mitochondria among HA and A172 cells. (**A**) HPLC−MS chromatograms of MitoQ at different concentrations. (**B**) The calibration curve of MitoQ within the concentration range of 1−1000 ng/mL. The concentrations of MitoQ in whole cells and the isolated mitochondria of HA (**C**) and A172 cells (**D**).

It is hypothesized that defects in mitochondrial function could be the reason why cancer cells rely on aerobic glycolysis for energy supply [31]. The mitochondrial structure and functions of cancerous cells differ from those of normal cells. Moreover, MitoQ targeting mitochondria is based on MMP and lipophilicity [32]. Therefore, the enrichment of MitoQ within cancerous and normal cells could be different. The concentrations of MitoQ in isolated mitochondria and whole cells of HA and A172 cells were determined using HPLC-MS to evaluate the mitochondria-targeted ability of MitoQ. The HPLC-MS calibration curve (y = 3446.7x − 9869.8, R^2^ = 0.9999) for MitoQ had been established at concentrations of 1, 10, 100, and 1000 ng/mL and subsequently became feasible and linear (R^2^ > 0.99) (Figure 2A,B). The concentrations of MitoQ in whole cells and the isolated mitochondria of HA cells were higher than those in A172 cells, demonstrating that MitoQ was more easily enriched in normal cells (Figure 2C,D). Treatment of HA with MitoQ for 2 h resulted in a >80% enrichment of MitoQ in mitochondria, indicating that MitoQ could be effectively enriched within mitochondria of normal cells (Figure 2C).

### 3.3. MitoQ-Induced PMMP in Normal Cells Was Higher than That in Tumor Cells

**Figure 3:**
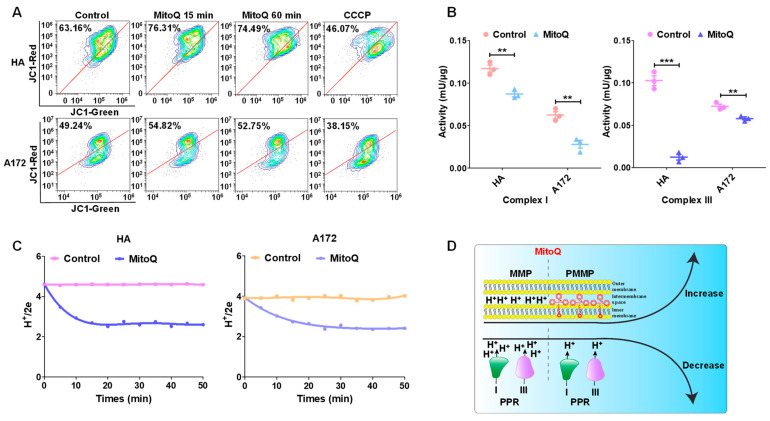
Construction of PMMP using MitoQ. (**A**) Fluorescence of HA and A172 cells stained using JC-1 was ascertained through flow cytometry. (**B**) The activities of respiratory chain complexes I and III associated with proton production were determined with commercial kits. (**C**) The PPR was detected through a fast-responding pH electrode system after MitoQ treatment. (**D**) The schematic diagram of mitochondrial status changes after MitoQ treatment. Each experiment was conducted at least three times. All the data were presented as mean ± SEM. Error bars represent SEM, and statistical significance between groups was analyzed using an unpaired *t*-test. ** *p* < 0.01; *** *p* < 0.001.

HA and A172 cells treated with MitoQ were stained using JC-1 and analyzed with flow cytometry to determine changes in MMP. Through treatment with MitoQ for 15 min, the MMP of HA cells increased by 13.15%, whereas that of A172 cells only increased by 5.58% (Figure 3A). The increase could be associated with the HPLC-MS results that showed that MitoQ was enriched in the mitochondria of HA cells at a much higher level than A172 cells (Figure 2C,D). In the meantime, the activities of respiratory chain complexes I and III and the PPR were restrained by MitoQ (Figure 3B,C). Therefore, when MitoQ adsorbed to the inner mitochondrial membrane and remained the cationic moiety in the intermembrane space, the result was the addition of a large number of positive charges to protons. The balance of MMP was maintained by MitoQ attenuating the activity of respiratory chain complexes I, III, and IV (which are associated with proton generation) and decreasing the PPR (Figure 3C). MitoQ successfully constructed PMMP by proton displacement with exogenous positive charges based on our previous hypothesis (Figure 3D) [13]. The MMP and respiratory chain are important for mitochondrial respiration [33]. Therefore, MMP and respiratory chain complex dysfunction could lead to abnormal mitochondrial respiration.

### 3.4. PMMP Disrupted Energy Metabolism in HA Cells

Metabolomics can offer insights into the cellular processes in response to stimuli or interactions, for the metabolome is considered the closest phenotype representation [34,35]. Untargeted metabolomics was performed on HA and A172 cells treated with MitoQ further to determine the effect of MitoQ on energy metabolism. 

**Figure 4:**
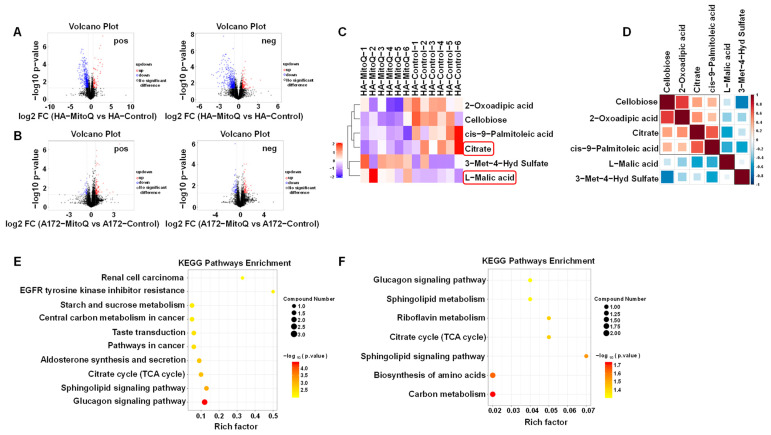
MitoQ disrupted the TCA cycle in HA cells. Effect of MitoQ on metabolite expression in HA (**A**) and A172 cells (**B**), *n* = 6. The Fold Change (FC) analysis and *t*-test were utilized in volcano plot analysis to screen the potential metabolites. The red dots indicate differential metabolites with FC > 1.5, and *p*-value < 0.05, the blue dots reveal differential metabolites with FC < 0.67 and *p*-value < 0.05, and the black dots depict no significant difference. (**C**) Influence of MitoQ on citrate and L-malic acid expression within HA cells. (**D**) The expression of citrate and L-malic acid were negatively correlated within HA cells. KEGG analysis of metabolites pathway after MitoQ treatment using HA (**E**) and A172 cells (**F**).

The global orthogonal partial least-squares-discriminant analysis (OPLS-DA) model revealed a clear separation between the MitoQ-treated and control groups in positive and negative ion modes (Figure S1). The permutation testing was performed on the quality of the model and revealed that the model was not over-fitted (Figure S2). The volcano maps showed that more differential metabolites appeared in MitoQ-treated HA cells than in MitoQ-treated A172 cells compared to the control group (Figure 4A,B), indicating that MitoQ had a more significant effect on HA than A172 cells. 

The expressions of citrate and L-malic acid, the primary metabolites of the TCA cycle [36], were altered after HA cells were treated with MitoQ (Figure 4C and Figure S3). Moreover, their expression changes were negatively correlated (Figure 4D). The citrate expression was down-regulated, while L-malic acid was up-regulated. The results displayed that the TCA cycle of HA cells could be disturbed after treatment with MitoQ. The KEGG enrichment pathway map results also revealed that the TCA cycle of MitoQ-treated HA cells significantly differed from the control group (*p* = 0.0014) (Figure 4E). MitoQ also had a slight effect on the TCA cycle of the A172 cells (*p* = 0.0378) (Figure 4F), only down-regulating the expression of citrate (Figure S3). Since tumor cells primarily rely on aerobic glycolysis for energy supply, a minimal change in the TCA cycle of A172 cells does not affect the energy supply of A172 cells. As shown in Figure 3 and Figure 4, the main target of the PMMP constructed using MitoQ was mitochondrial respiration. Therefore, it could effectively disrupt the energy metabolism and subsequent energy supply of normal cells, but it was ineffective against tumor cells. 

### 3.5. MitoQ Induced Autophagy in HA Cells through the AMPK/mTOR Pathway

**Figure 5:**
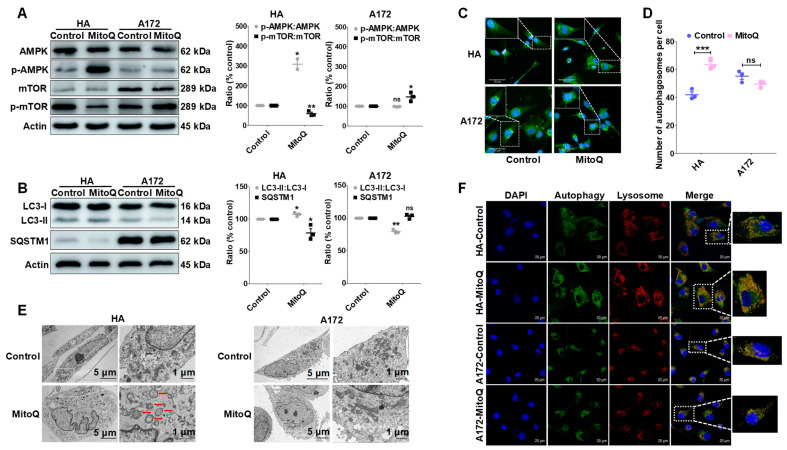
MitoQ induced autophagy in HA cells through the AMPK/mTOR pathway. (**A**) The phosphorylation of AMPK and mTOR in HA and A172 cells were evaluated using Western blotting after treatment with MitoQ. (**B**) The conversion of LC3-I to LC3-II and the protein levels of SQSTM1/p62 were analyzed through Western blotting. The autophagosomes induced by MitoQ were visualized (**C**) and determined (**D**) by the high content imaging system. (**E**) Transmission electron microscopy was utilized to observe the autophagic vacuoles. (**F**) The autophagosome–lysosome fusions were visualized through confocal microscopy. Representative images were provided as indicated. Each experiment was conducted at least three times. All the data are presented as mean ± SEM; error bars represent SEM. Statistical significance between the groups was analyzed using unpaired *t*-test. * *p* < 0.05; ** *p* < 0.01; *** *p* < 0.001; “ns” represents no statistical difference.

The cellular energy sensor AMPK is activated by energy deprivation. Thus, its activity is related to cellular energy metabolism levels and autophagy [37]. Based on the Western blotting analysis results, AMPK was activated by enhancing the phosphorylation of AMPK after treatment using MitoQ in HA cells. Moreover, the phosphorylation of mTOR was significantly downregulated in HA cells (Figure 5A). However, the AMPK/mTOR pathway in A172 cells was not activated after MitoQ treatment (Figure 5A). Then, the protein levels of the autophagy markers MAP1LC3/LC3 (microtubule-associated protein 1 light chain 3) and SQSTM1/p62 [38] were tested in cells treated with MitoQ compared to control. A significant increase was observed in the LC3-II: LC3-I ratio levels, with a significant decrease in the protein levels of SQSTM1/p62 in HA cells treated with MitoQ. For A172 cells, MitoQ pre-treatment downregulated the LC3-II: LC3-I ratio level and did not affect SQSTM1/p62 (Figure 5B). Following the inhibition of MitoQ-induced autophagy flux by chloroquine (CQ), an alkalinizing agent blocking autophagosome fusion with the lysosome [39], the expression of LC3-II was further assessed via Western blotting. As illustrated in Figure S4, the level of LC3-II in HA cells with MitoQ + CQ treatment was significantly higher than that of the MitoQ and CQ groups. The results indicated that CQ’s inhibition of MitoQ-induced autophagy flux resulted in an increase in the expression level of LC3-II. The findings indicated that MitoQ was capable of stimulating autophagy flux in HA cells. In addition, the Autophagy Assay Kit staining revealed a significant increase in the number of autophagosomes in HA cells treated with MitoQ. However, the number of autophagosomes in A172 cells treated with MitoQ did not change (Figure 5C,D). The same results were also observed by TEM (Figure 5E). Furthermore, there were more autophagosome–lysosome fusions in MitoQ-treated HA cells than in control. There was no difference between the two groups of A172 cells among autophagosome–lysosome fusions (Figure 5F). The results indicated that MitoQ stimulated autophagy flux through the AMPK-mTOR pathway in HA cells.

### 3.6. Protective Effect of MitoQ on Normal Cells against Radiation

**Figure 6:**
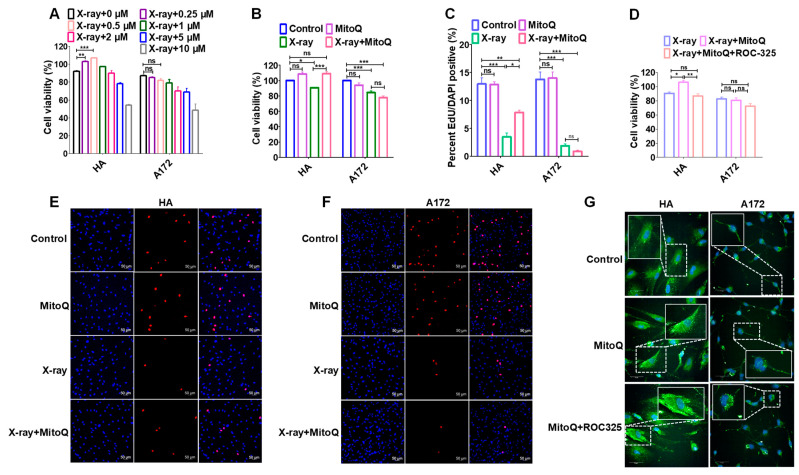
The protective effect of MitoQ on HA cells against X-ray radiation. (**A**) Effects of different concentrations of MitoQ on HA and A172 cells during X-ray radiation were detected through CCK8 assays. (**B**) MitoQ at a concentration of 0.5 μM promoted the proliferation of HA cells during 4 Gy X-ray radiation in CCK8 assays. (**C**) EdU assay demonstrated that MitoQ could protect HA cells from damage using X-rays. Typical photos of the EdU assay were captured with confocal microscopy (**E**,**F**). (**D**) Autophagy inhibitor ROC-325 destroyed the protective effect of MitoQ on HA cells. (**G**) ROC-325 restrained the autophagy flux. Representative images were provided as indicated. All the data are presented as mean ± SEM from at least three independent experiments, and error bars represent SEM. Statistical significance between groups was analyzed using one-way ANOVA, * *p* < 0.05; ** *p* < 0.01; *** *p* < 0.001; “ns” represents no statistical difference.

The protective effect of MitoQ on normal cells by inducing autophagy during radiation was detected using CCK8 and EdU assays. MitoQ protected HA cells from X-ray exposure in a dose-dependent manner at 0–0.5 μM but did not affect A172 cells in CCK8 assays (Figure 6A). Mito effectively protected the survival of HA cells but did not affect the proliferation inhibition of A172 cells under 4Gy X-ray radiation (Figure 6B). EdU assays also demonstrated that MitoQ could protect HA cells from damage by X-rays, but not the A172 cells (Figure 6C,E,F). MitoQ protected normal cells not only under low linear energy transfer (LET) radiation but also under high LET radiation. CCK8 and EdU assays revealed that MitoQ could protect HA cells from damage by 2Gy carbon ions, which has a high LET [40], but did not affect the inhibitory effect of carbon ions on A172 cells (Figure S5). ROC-325 is a novel autophagy inhibitor that can lead to the deacidification of lysosomes, accumulation of autophagosomes, and the disruption of autophagy flux [41]. When MitoQ-induced autophagy flux was blocked within HA cells using ROC-325, the number of autophagosomes was significantly enhanced compared to that treated using MitoQ alone (Figure 6G). Correspondingly, the protective effect of MitoQ on HA cells was destroyed by ROC-325 (Figure 6D). Therefore, MitoQ-constructed PMMP-inducing autophagy played an essential role during normal cellular radioprotection. 

### 3.7. MitoQ Protected Normal Tissue against X-rays in Mice Bearing Orthotopic Glioma 

**Figure 7:**
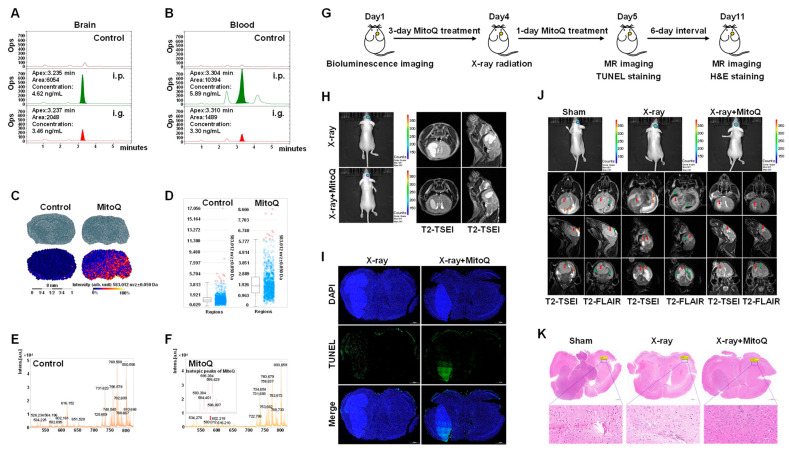
Protective effect of MitoQ on normal tissues against X-rays among mice bearing orthotopic glioma. HPLC-MS chromatograms of MitoQ inside the brain (**A**) and blood (**B**) of both the i.p. and ig. group, *n* = 5. (**C**) MALDI-TOF-MS was used to visualize the spatial distribution of MitoQ on the surface of brain tissue sections. The intensity box blot (**D**) and the representative MALDI-TOF-MS mass profile (583 m/z) (**E**,**F**) of MitoQ on the surface of the brain tissue sections were observed. The isotopic peaks of the MitoQ procured from MALDI-TOF-MS were also shown (**F**). (**G**) Schematic diagram illustrating the experimental design. Bioluminescence imaging was used to determine the tumor size of the nude mice bearing luciferase-positive U87MG orthotopic brain tumors on Day 1 (before treatment). The mice were intraperitoneally injected using MitoQ (10 mg/kg/day) for four days (Day 2–Day 5). The mice received 16 Gy X-ray whole-brain radiation on Day 4, two hours after MitoQ administration. MR imaging and TUNEL staining were performed on Day 5, and MR imaging and H&E staining were performed on Day 11. (**H**) The tumors were evaluated by bioluminescence before treatment, and MRI signals in the brain were monitored 24 h post-X-ray radiation. (**I**) TUNEL-stained sections were obtained at 24 h post-X-ray radiation. (**J**) The tumors were evaluated using bioluminescence before treatment, and MRI signals in the brain were detected seven days post-X-ray radiation. Red arrows indicate tumors, orange indicates edema, and green indicates hydrocephalus. (**K**) H&E staining of brain tissue on seven days post-X-ray irradiation. *n* = 5, Representative images were provided as shown.

The protective effect of MitoQ on normal cells was demonstrated in mice bearing orthotopic glioma. HLPC-MS and MALDI-TOF-MS imaging were performed to render the penetration and distribution of MitoQ in the brain. MitoQ was detected in the brain and blood of the i.p. and i.g. groups using HLPC-MS, and the concentrations of MitoQ in the brain and blood in the i.p. group were higher than that in the i.g. group (Figure 7A,B). Therefore, i.p. injection was selected for MitoQ administration. MALDI-TOF-MS visualized the spatial distribution of MitoQ on the surface of brain tissue sections (8 μm). A high level of MitoQ was witnessed in the brain tissue sections, indicating that MitoQ could penetrate the brain tissue (Figure 7C,D). A portion of the MALDI-TOF-MS spectrum within the mass ranges of 0–820 m/z obtained from brain tissue sections of the control and MitoQ-treated group also revealed that the representative MALDI-TOF-MS mass profile (583 m/z) of MitoQ was detected in brain tissue sections of MitoQ-treated group, and not in the control group (Figure 7E,F). These data depicted that MitoQ could be distributed in the brain.

Next, we determined whether MitoQ protected against structural damage to normal tissue due to X-rays using the nude mice bearing luciferase-positive U87MG orthotopic brain tumors. The experimental design was shown on Figure 7G. We established orthotopic brain tumors and assessed the tumors using bioluminescence before treatment (Figure 7H,J). MRI signals in the brain were monitored and analyzed 24 h and seven days post-X-ray radiation. MitoQ reduced the edema in normal brain tissue after 24 h of X-ray radiation, as the MR images demonstrated that the normal brain tissue of the mice in the X-ray group was brighter than that of the mice in the X-ray + MitoQ group (Figure 7H). The TUNEL-stained sections revealed that MitoQ promoted apoptosis inside brain tumors 24 h post-X-ray radiation (Figure 7I). After seven days of X-ray radiation, MRI signals showed that MitoQ could reduce X-ray treatment-induced edema and hydrocephalus of normal tissue inside the mouse brain without affecting the X-ray tumor treatment compared to the X-ray group (Figure 7J). We also observed that normal brain tissue cells in the X-ray group were less compacted than in the X-ray + MitoQ group. It was observed that the normal brain tissue in the X-ray group was surrounded by more edema and hydrocephalus than in the X-ray + MitoQ group (Figure 7K).

## 4. Discussion

RT is one of the essential treatment methods against malignant tumors, but the damage to normal tissues is the fundamental challenge for RT [42]. In survivors, RT-induced secondary tumors, cognitive dysfunction, cardiac toxicity, gastrointestinal toxicity, radiation dermatitis, etc., are common [4]. In 1902, the first radiation-related cancer, a radiation-induced secondary primary skin cancer, was discovered [43]. Subsequently, the Gustave Roussy Institute conducted a long-term follow-up of 7711 breast cancer patients between 1954 and 1983 and observed that approximately 77% of the patients with secondary tumor recurrence had a history of RT [44]. With the increasing number of radiation-induced secondary primary tumors (such as leukemia, lymphoma, brain tumors, etc.), malignant tumors are gradually recognized as a long-term effect of radiation [45]. Thus, the critical goal and principal challenge of RT is the cancellation of tumors without any concurring injuries to the surrounding tissues and organs. Modern RT methods include three-dimensional conformal, and intensity-modulated radiotherapy (IMRT) could reduce, but not eliminate, the incidence and severity of toxicities [46]. This provides a strong rationale for adding radio-protective agents. However, the only small radio-protector molecule approved by FDA, amifostine, has severe adverse effects limiting its clinical use [47]. Many antioxidants ameliorate or prevent the side effects of RT, but some even potentially promote cancer development and metastasis [7,8,9,10]. Therefore, the lack of precision of radiation protection and serious side effects have always been the main reasons restricting the development and application of radiation protection drugs in cancer radiotherapy. 

In this study, MitoQ successfully constructed PMMP through proton displacement with exogenous positive charges by adsorbing to the inner mitochondrial membrane and remaining the cationic moiety in the intermembrane space. The activities of respiratory chain complexes I and III were restrained, resulting in reduced PPR (Figure 3). MitoQ-constructed PMMP selectively induced autophagy within normal cells but not tumor cells (Figure 5) by interfering with the energy metabolism of normal cells (Figure 4). Autophagy can serve as a protective mechanism by executing the degradation and elimination of damaged organelles and providing energy for cellular renovation during stress conditions, including X-ray radiation [48,49]. For instance, autophagy is promoted to recycle unnecessary organelles to enhance amino acid availability in response to starvation [50]. Endothelial dysfunction plays an essential role in liver injury. Autophagy can maintain endothelial phenotype and protect liver sinusoidal endothelial cells from oxidative stress during the early phases of liver disease [51]. In addition, autophagy is a critical protective pathway in neurons, which relies on autophagy to preserve cytoplasmic homeostasis [52]. In this study, MitoQ-constructed PMMP selectively induced protective autophagy to exert specific protection for normal brain cells and tissues (Figure 6 and Figure 7). This occurred because the difference in energy metabolism phenotypes of normal and tumor cells led to the difference in the response of the two cell types due to radiation, and this difference is precisely the entry point for specific radioprotection of normal cells.

The Warburg effect has been proposed as one of the malignant phenotypes of tumors, establishing a basis for our understanding of tumors and precision therapy [53]. For instance, an unrevealed molecular function of corannulene buckybowl glycoconjugates is to selectively annihilate tumors by targeting the cancer-specific Warburg effect [54]. Basic leucine zipper and W2 domain-containing protein 1 (BZW1) promotes tumor growth by facilitating aerobic glycolysis and serves as a therapeutic target for pancreatic cancer patients [55]. However, the value of the presence of the Warburg effect in tumor cells has never been unearthed in radioprotection. In this study, we exploited the cancer-specific aerobic glycolysis (Figure 1) to detect an entry point for precise radioprotection of normal cells. As is known, tumor treatment has entered the “Precision Era.” Thus, the “Precision Era” includes “Precision Treatment” and “Precision Protection.” In the precise treatment of tumors, it is fundamental to detect targeted drugs through genetic screening. Combined with our work, we suggest that the differences in energy metabolism during tumor treatment provide a precise strategy to facilitate normal tissue radiation protection. Therefore, energy metabolism screening should be paid attention to during tumor treatment.

## 5. Conclusions

In summary, our study demonstrated that MitoQ-constructed PMMP selectively protects normal cells and tissues in glioma RT without affecting its efficacy by inducing autophagy in normal cells by regulating the cellular energy supply. These findings provide insights into the targeted protection of MitoQ-induced PMMP on normal tissues due to the different energy phenotypes between tumor cells and normal cells. It supports a preclinical rationale to broaden the clinical evaluation of energy metabolism screening during tumor treatment. These findings also provide solid evidence to support our pioneer work on pseudo-mitochondrial membrane potential for practical application.

## Data Availability

The data generated in this study are available within the article and its supplementary data files.

## Supplementary Materials

The following supporting information can be downloaded at: https://www.mdpi.com/article/10.3390/antiox12020453/s1.
