# Supplementary material for: Mitochondrial-Targeted Antioxidant MitoQ-Mediated Autophagy: A Novel Strategy for Precise Radiation Protection
Source: Antioxidants (Basel). 2023 Feb 10;12(2):453. doi: 10.3390/antiox12020453 (PMC9952602; doi:10.3390/antiox12020453)
Supplement: Supplementary file 1 [file antioxidants-12-00453-s001.zip › antioxidants-2129034-supplementary.pdf]

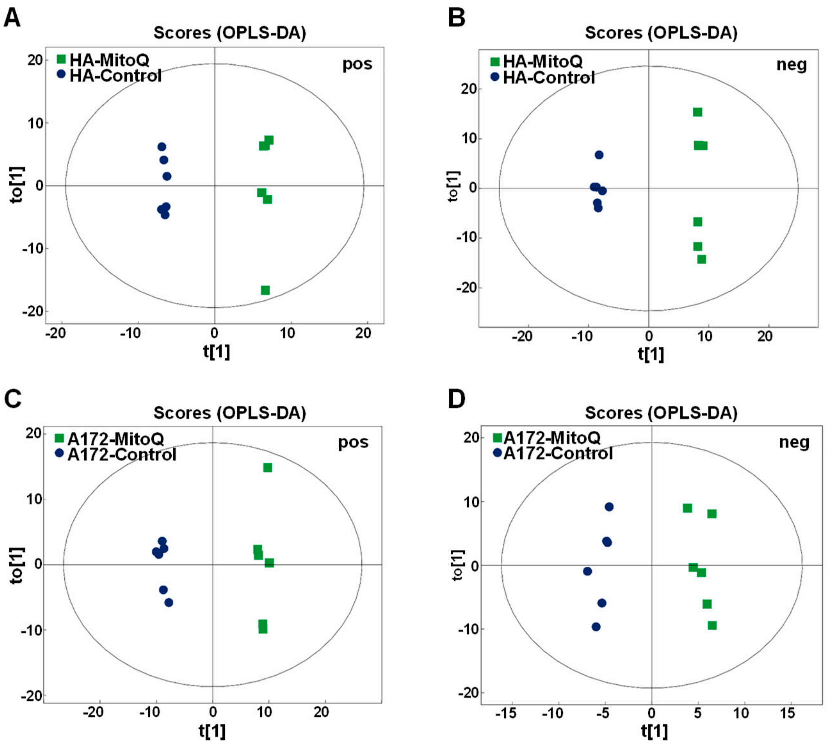

Figure S1. OPLS-DA score plot revealed an excellent separation between MitoQ-treated and control groups. OPLS-DA score plot between the MitoQ-treated HA cells and control in positive (A) and negative (B) ion mode. OPLS-DA score plot between MitoQ-treated A172 cells and control among positive (C) and negative ions mode (D).

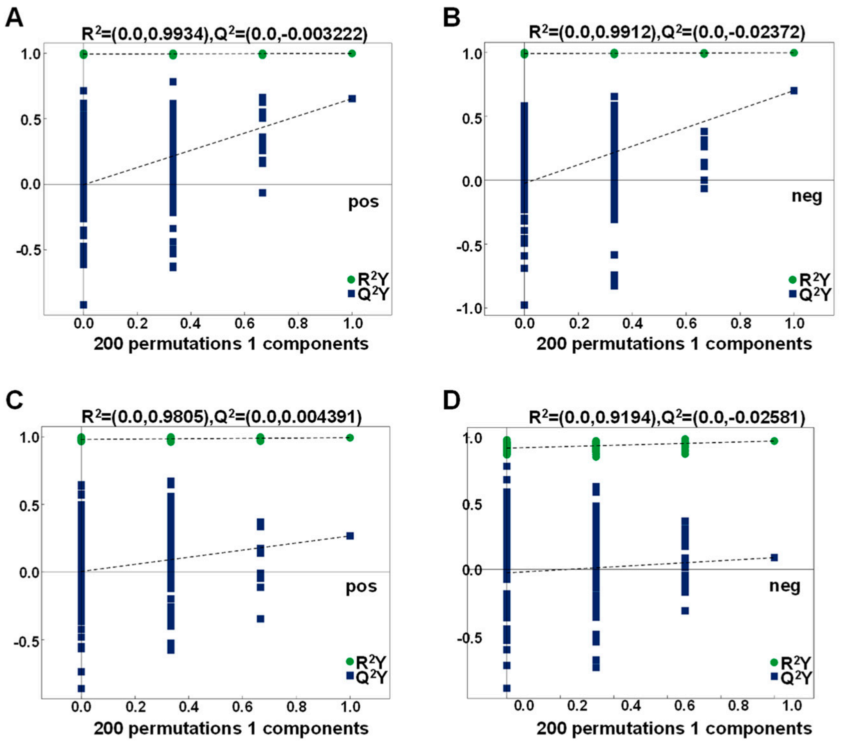

Figure S2. Permutation testing depicted that the model was not over-fitted. Permutation testing between MitoQ-treated HA cells and control within positive ion (A) and negative ion mode (B). Permutation evaluation between MitoQ-treated A172 cells and control within positive ion (C) and negative ion mode (D).

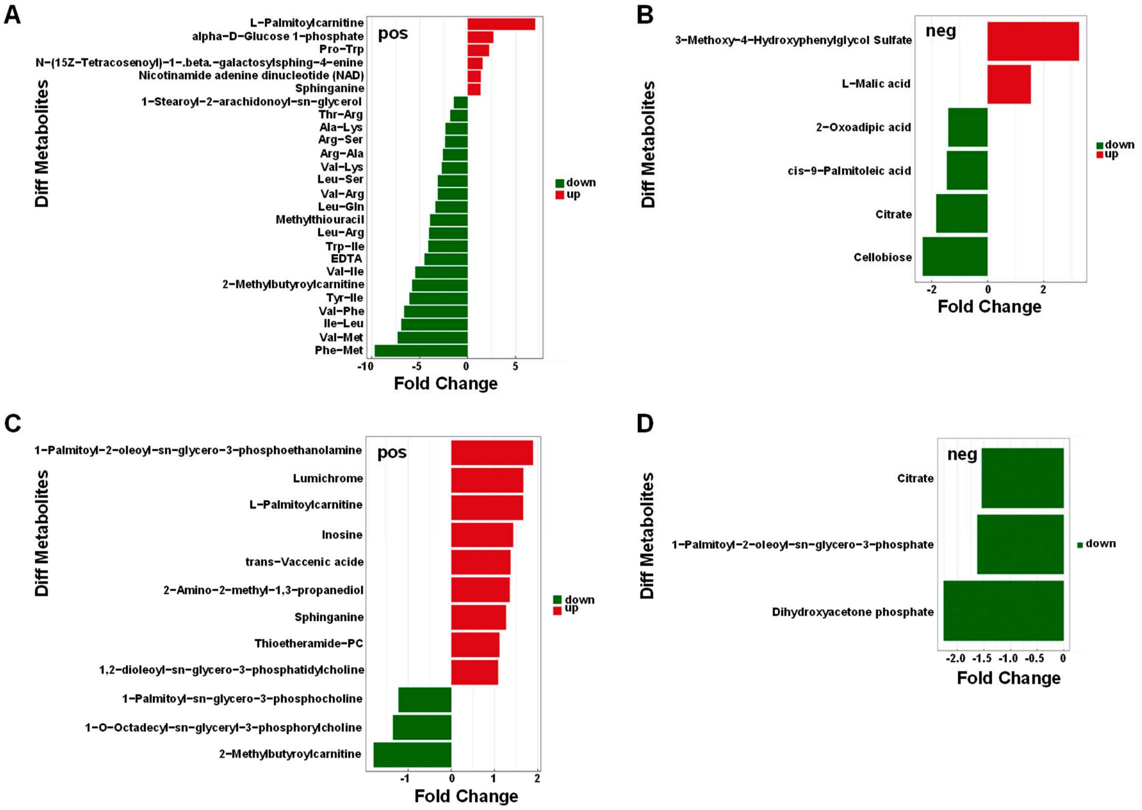

Figure S3. Significantly altered metabolites with VIP > 1 and p-value < 0.05. Significantly altered metabolites of HA-MitoQ vs. HA-Control within positive ion (A) and negative ion mode (B). Significantly altered metabolites of A172-MitoQ vs. A172-Control in the positive (C) and negative ion mode (D).

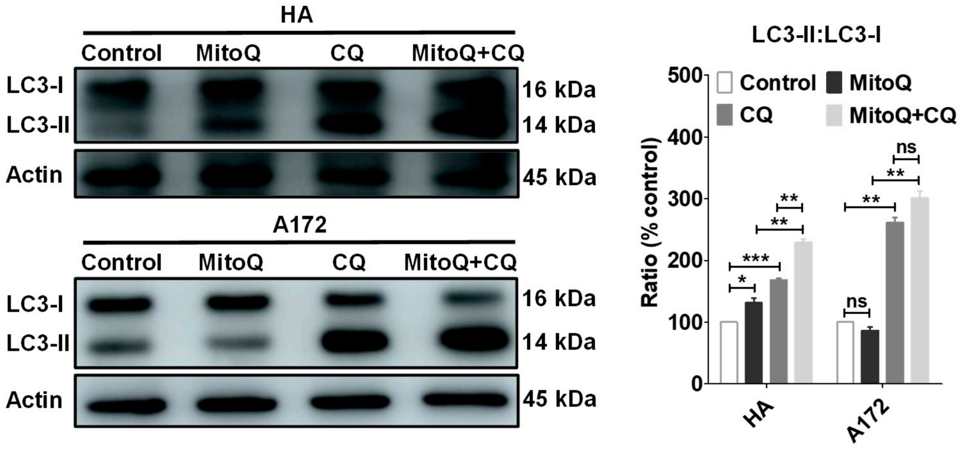

Figure S4. The LC3-II expression was assessed by western blotting after CQ inhibited the autophagy flux induced by MitoQ. All the data are presented as mean ± SEM; error bars represent SEM. Statistical significance between the groups was analyzed using unpaired t-test. *p < 0.05; **p < 0.01; ***p < 0.001; "ns" represents no statistical difference.

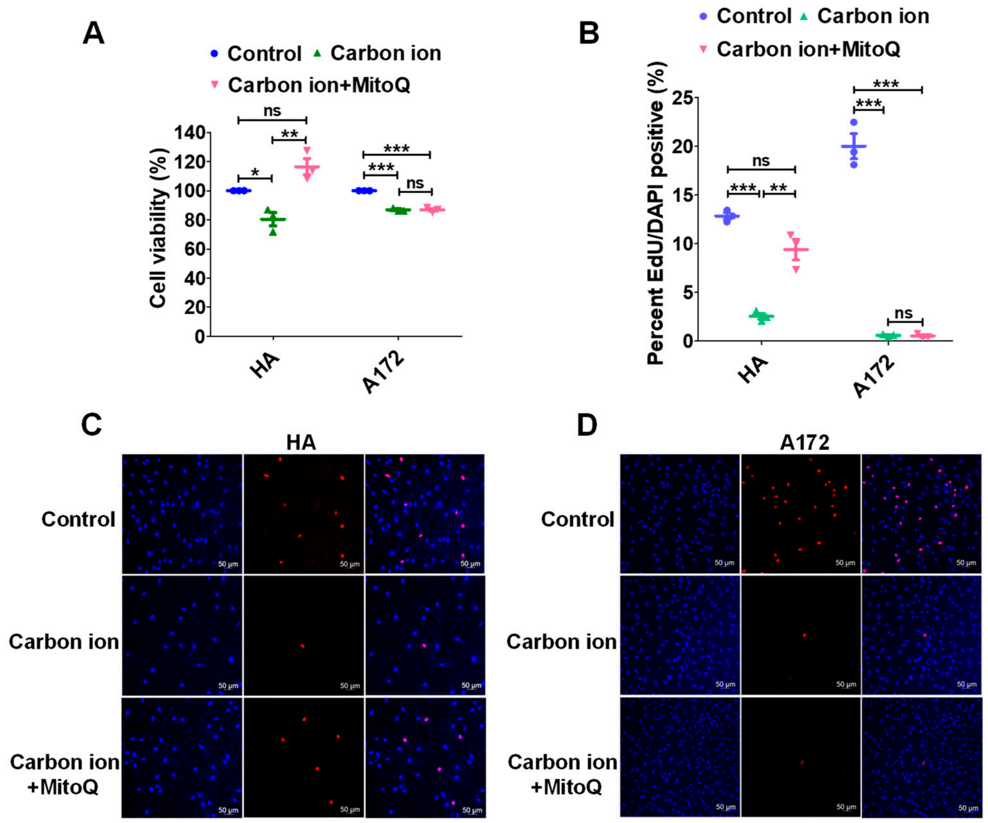

Figure S5. The protective effect of MitoQ over HA cells against carbon ion radiation. (A) MitoQ promoted the proliferation of HA cells during 2 Gy carbon ion irradiation in the CCK8 assays. (B) EdU assay demonstrated that MitoQ could protect HA cells from damage due to carbon ions. Typical photos of the EdU assay were captured using confocal microscopy (C and D). Representative images were provided as indicated. All the data are presented as mean ± SEM from at least three independent experiments, and error bars represent SEM. Statistical significance between groups was analyzed using one-way ANOVA, *p < 0.05; **p < 0.01; ***p < 0.001; "ns" represents no statistical difference.
